# Exploring the variations in death anxiety among oncology nurses in China: a latent class analysis

**DOI:** 10.1186/s12904-023-01282-6

**Published:** 2023-11-09

**Authors:** Xian Chen, Mengyu Su, Anne Arber, Chengping Qiao, Jinfeng Wu, Cuihua Sun, Dan Wang, Hui Zhou, Zhu Zhu

**Affiliations:** 1 Women’s Hospital of Nanjing Medical University, Nanjing Maternity and Child Health Care Hospital), Nanjing, Nanjing, 210004 China; 2 School of Nursing, Nanjing Medical University, Nanjing, 211166 China; 3 School of Health and Sciences, Faculty of Health and Medical Sciences, University of Surrey, Guildford, GU2 7XH UK; 4 Geriatrics Department, The First Affiliated Hospital of Nanjing Medical University, Nanjing, 210029 China; 5 Jiangsu Nursing Association, Nanjing, 210008 China; 6 Oncology Department, The Affiliated Hospital of Xuzhou Medical University, Xuzhou, 221002 China

**Keywords:** Palliative care, Death anxiety, Oncology nurses, China, Latent class analysis

## Abstract

**Background:**

Various factors have been found to be associated with high levels of death anxiety experienced by oncology nurses. The aim of this study was to use a person-oriented approach to examine the death anxiety patterns of Chinese oncology nurses and to analyze the differences in anxiety characteristics and their associated influencing factors.

**Methods:**

A cross-sectional survey regarding palliative care among registered oncology nurses was conducted in Jiangsu Province, China.Latent class analyses was applied to identify their patterns of death anxiety. The score of PCQN-C (The Chinese version of the Palliative Care Quiz for Nursing) and FATCOD-B-C (The Chinese version of the Frommelt Attitude Toward Care of the Dying scale), the demographic and working characteristics were further analyzed through covariance analysis (ANCOVA) and multivariate (or logistic) regression across the subgroups.

**Results:**

A two-potential-category model was selected based on the fit index. The results showed that 79% of oncology nurses belonged to the high pressure and pain group and 21% belonged to the low death anxiety group. The high pressure and pain group had significantly higher scores in the dimensions of emotion, stress and pain, time awareness, and cognition compared to the low death anxiety group. Factors influencing the high pressure and pain group included shorter working years, non-national or provincial oncology nursing specialists, non-national palliative care specialists, never discussing the topic of death with patients or family members, no palliative care related training, and PCQN and FATCOD scores.

**Conclusions:**

Our study suggests that oncology nurses’ death anxiety can be divided into two categories: low death anxiety and high stress pain, and certain factors, such as being female, having a short work experience, and lacking palliative care-related training, increase the likelihood of death anxiety.

## Introduction

Palliative care is a fundamental aspect of healthcare that aims to alleviate the suffering of patients and enhance their quality of life in the face of life-threatening illnesses [1]. In 2017, the National Health Commission of the People’s Republic of China issued the “Guidelines for Palliative Care Practice (Trial),“ which outlined the key principles and precautions for nurses in symptom management, psychological support, humanistic care, and palliative care delivery [2]. Therefore, oncology nurses play a crucial role in providing high-quality palliative care to patients with terminal cancer [3]. However, providing care to dying patients is not only challenging but also demanding work, which may increase anxiety among nurses, especially among those who are newly qualified [4–6]. The intense nature of palliative care work exposes oncology nurses to death and to those who are dying on a regular basis. This frequent exposure can provoke feelings of death anxiety. .

Death anxiety is an emotional response that arises from the apprehension of death, which can be real or imaginary and poses a threat to one’s life or the lives of those in close proximity [7]. Research indicates that more than half of nurses working in palliative care experience moderate to high levels of death anxiety [8]. Nurses who experience a heightened fear of death may manifest behavioral reactions such as avoidance, procrastination, or poor job performance, ultimately compromising their ability to deliver high-quality care to patients at the end of life [9]. Furthermore studies have shown that higher levels of death anxiety in nurses who provide palliative care services have been linked to increased burnout and decreased job satisfaction [4, 10]. This suggests that death anxiety not only affects their work-related quality of life but also impacts their emotional well-being and personal sense of fulfillment. Nurses experiencing significant death anxiety may struggle with managing the emotional challenges associated with caring for dying patients, leading to psychological distress and a diminished overall quality of life [11].

Various factors have been found to be associated with high levels of death anxiety experienced by oncology nurses, including their socioeconomic status, personal experiences, coping mechanisms, as well as job demands [12, 13]. In addition, studies have indeed highlighted the association between gender and death anxiety among healthcare professionals, including nurses. It has been observed that female nurses tend to report higher levels of death anxiety compared to their male counterparts [14, 15]. This gender difference may be influenced by a variety of factors, including societal expectations, cultural norms, and differences in coping styles. However, research has indicated that the duration of work experience can influence nurses’ levels of death anxiety. Nurses who have less experience or are newly qualified may exhibit higher levels of death anxiety, possibly due to limited exposure to end-of-life care situations and less confidence in managing the emotional challenges associated with caring for dying patients [6, 9]. When nurses have a positive and open emotional response, as well as a higher level of comfort and willingness to provide care for dying patients, there tends to be a decrease in their avoidance and fear of death [16]. Moreover, increased knowledge of palliative care has been found to lessen nurses’ death anxiety [6]. However, in China, this anxiety may be further amplified due to prevalent societal taboos regarding death. These cultural norms often hinder open communication about end-of-life care between oncology nurses and their patients [17]. For example, in many Chinese cultures, discussions around death are avoided due to a fear of inviting bad luck or misfortune. This cultural avoidance, coupled with societal attitudes towards death, enhances its perceived mystery, which can subsequently heighten nurses’ anxiety related to death [18].

Prior studies on death anxiety among nurses have primarily focused on exploring the relationship between the overall level of death anxiety and its associated influencing factors, disregarding the specific conditions and characteristics of individual death anxiety or the identification of internal heterogeneity [17, 19]. To address this gap, an individual-centered research approach is necessary to analyze the heterogeneity of death anxiety among oncology nurses. Latent Class Analysis (LCA) is a person-oriented approach that identifies different groups with unique characteristics within a population, with the goal of maximizing differences between groups while minimizing differences within groups. LCA is a widely accepted approach in the field of psychology, and the accuracy and effectiveness of LCA can be evaluated using objective statistical indicators [20]. This study focused on the types of death anxiety of Chinese oncology nurses and used LCA to analyze the differences in anxiety characteristics among different classes and their associated influencing factors, providing nurse managers and policymakers a reference for targeted and individualized interventions.

## Methods

### Design and sample

A quantitative cross-sectional survey was conducted using an online survey method to investigate the prevalence of death anxiety and its associated factors among registered oncology nurses in Jiangsu Province, China. The Jiangsu Nursing Association played a valuable role in facilitating the survey administration and reaching out to the registered oncology nursing in Jiangsu Province. To ensure the representativeness of the cross-sectional study, a regional stratified sampling method was utilized to obtain a representative sample of oncology nurses across the 13 regions of Jiangsu Province as described in detail previously [21]. All registered oncology nurses working in the inpatient ward of the hospitals for at least six months were eligible to participate in the study. To ensure the representativeness of the sample, regions with fewer than 1,000 responses included all valid answers, while regions with over 1,000 responses randomly selected 1,000 valid answers. The survey was conducted using a mobile-based application called “Wenjuanxing”. Prior to beginning the survey, participants were provided with written explanations about the study’s purpose and the questionnaire’s requirements. Participants were also informed that this study was completely voluntary and they could withdraw from the study at any time, and electronic consent was obtained from all participants. Data were collected took place from May 10 to May 30, 2021. Ethical approval was obtained from the Jiangsu Nursing Association and the Women’s Hospital of Nanjing Medical University (approval number: 2021-KY-025).

### Instruments

#### Demographic data

The participants’ demographics, nursing professional background and palliative care experience were the first part of the questionnaire. The demographic characteristics included gender, age, marital status, and religious beliefs. Nursing professional background included hospital classification, working years, nurse position, whether qualified as a palliative care nursing specialist.

### The Chinese version of the Templer Death Anxiety Scale (CT-DAS)

The Templer Death Anxiety Scale (T-DAS) was developed by Professor Donald Templer to measure the level of death anxiety [22]. We used the Chinese version of the Templer Death Anxiety Scale (CT-DAS) that was cross-culturally debugged by Yang [23]. The CT-DAS has been used among Chinese nurses and has shown good reliability and validity [17]. The scale contains 15 questions which are divided into four dimensions, including emotion (measuring one’s emotions regarding experiences and perspectives of death), stress and distress (measuring one’s stress and distress brought by death), extinguishment of time (concerning one’s realization of time and life), and cognition with life and death (regarding one’s cognitive recognition with life and death). Nine of the 15 items are keyed “true” and six are keyed “false”. The overall scores are 0–75 points and a score of 35 points is used as a standard threshold for high death anxiety. The higher the score, the higher the death anxiety level. The Cronbach’s α and test-retest stability of the Chinese version was 0.83 and 0.71, respectively.

### The Chinese version of the Palliative Care Quiz for Nursing (PCQN-C)

Nurses knowledge of palliative care was measured by Palliative Care Quiz for Nursing (PCQN), which is originally designed by Ross [24]. The Chinese version of PCQN was used in previous studies and had been confirmed with good reliability and validity [25]. The PCQN-C consists of 20 questions, divided into three dimensions: philosophy and principles of palliative care (4 items), pain and symptom management (13 items), and psychosocial and spiritual care (3 items). Each question has three possible responses including “true”, “false” and “do not know”; 1 point is counted for a correct answer, 0 points for incorrect answers or do not know. The total score is ranged from 0 to 20. The higher the score, the better the knowledge of palliative care. The test-retest reliability of the Chinese version was 0.782 and internal consistency reliability is 0.758.

### The Chinese version of the Frommelt Attitude Toward Care of the Dying scale (FATCOD-B-C)

The Frommelt Attitude Toward Care of the Dying scale (FATCOD-B) was used to assess the attitude of palliative care among oncology nurses [26]. Previous studies found the FATCOD-B-C to be a reliable and valid measure of attitudes towards end-of-life care among Chinese healthcare workers [27, 28]. The 29 items on the FATCOD-B-C scale are divided into six subsets: attitude toward the interests of the dying person (6 items), attitude toward caring for the dying person (6 items), attitude toward the necessity of family support (5 items), attitude toward communication with the dying person (5 items), attitude toward fear of caring of dying person (3 items), and attitude toward caring for the dying persons families (4 items). The overall score ranges from 29 to 145. The higher the scale score, the more positive nurses’ attitude. The Cronbach’s α coefficient of the scale was 0.796, the Cronbach’s α coefficient of six subscales was 0.610–0.863, and the retest reliability of the scale was 0.959, and the content validity was 0.920.

### Statistical analysis

LCA was used to explore latent class of death anxiety. LCA models with 2 to 3 subgroups were fitted and compared to determine the optimal number of death anxiety subgroups. Combining model fitting indexes were taken into consideration, including Akaike information criterion (AIC), Bayesian information criterion (BIC), sample-size adjustment BIC (aBIC), the Lo–Mendell–Rubin likelihood ratio test (LMR-LRT), Bootstrapped likelihood ratio test (BLRT), and Entropy. The smaller the AIC, BIC, and aBIC, the better the model fit. Entropy is valued from 0.00 to 1.00, and the closer to 1.00, the more accurate the classification. After examining the best-fit model of latent classes, the data were further adjusted by demographic characteristics, compared the score of PCQN-C and FATCOD-B-C through analysis of covariance (ANCOVA) and multivariate (or logistic) regression across the subgroups.

The measurement data that follow the normal distribution were expressed as Mean ± Standard Deviation, and the intergroup comparison used analysis of variance. The nonnormal distribution of measurement data was expressed in median and quartile spacing, and the intergroup comparison was based on Kruskal-Wallis H test. The counting data were expressed in frequency and percentage, and the chi-square test was used for inter-group comparison. Statistical analysis was conducted using SAS version 9.4 and Mplus version 8.3. Statistical significance was set at *p* < 0.05 with two-sided tests.

## Results

### Description of the participants

In this study, a total of 5256 registered oncology nurses participated voluntarily. The research employed an online survey methodology, and notably, there were no instances of withdrawal or dropout among the participants. Participation was entirely motivated by the nurses’ willingness to contribute to our research objectives, and as such, no rewards or incentives were provided. The demographic characteristics of the participants are shown in Table 1. Among the 5256 registered oncology nurses, 98.23% were women, 70% were less than 35 years old, 38.68% had worked for 5–10 years, only 2.83% were national or provincial oncology nursing specialists, 2.17% were national palliative care specialists, 60.67% of oncology nurses had discussed the topic of death with patients or their family members, and approximately 23.52% of them had been trained with palliative care-related courses.

**Table 1:** Characteristics of the participants

Characteristic	Total number	(Percentage, %)
**Gender**		
Male	93	1.77
Female	5163	98.23
**Age**		
<35	3679	70
35–50	1455	27.68
>50	122	2.32
**Working years**		
<5	1728	32.88
5–10	2033	38.68
>10	1495	28.44
**Oncology nursing specialist**		
Yes	149	2.83
No	5107	97.17
**Palliative care specialist**		
Yes	114	2.17
No	5142	97.83
**Marital status**		
Single or divorced	1449	27.57
Married	3807	72.43
**Level of nursing job**		
Junior level	2722	51.79
Medium level or senior level	2534	48.21
**Experience of discussing death**		
Yes	3189	60.67
No	2067	39.33
**Attended the Palliative training Course**		
Yes	1236	23.52
No	4020	76.48
**Hospital classification**		
Tertiary hospital	3706	70.51
Secondary and Primary hospital	1550	29.49
**Personal beliefs**		
Yes	451	8.58
No	4805	91.42

### Description of latent classes of nurses’ death anxiety

In this study, two different potential category models were fitted, and the results of the adaptation index were shown in Table 2. The AIC and aBIC decreased as the number of potential classes in the model increased. When the three potential classes were retained (model 3), the Entropy < 0.8 and P > 0.05 for the LMR test which did not meet the conditions, so model 3 was excluded. Considering comprehensively, the LCA model of two potential categories was selected in this study in which Entropy was 0.812, *p*<0.05 for the LMR and BLRT tests. In the end, there were 1095 participants in class 1, accounting for 20.83%, and 4161 participants in class 2, accounting for 79.17%. The average probability of the two potential categories belonging to their potential categories was 96.07% and 84.53%, respectively, indicating the classification results of the two potential categories model were reliable.

**Table 2:** The results of the adaptation index of potential category models

Model	Potential category	AIC	aBIC	Entropy	LMR	BLRT	Classified probability
Model 1	1	47798.17	47835.458	—	—	—	—
Model 2	2	47675.67	47726.515	0.812	< 0.001	< 0.001	C1 = 20.83%
							C2 = 79.17%
Model 3	3	47607.85	47668.858	0.737	0.09	< 0.001	C1 = 8.05%
							C2 = 1.47%

Note: AIC, Akaike information criterion; aBIC, sample-size adjusted BIC; LMR, Lo-Mendell-Rubin; BLRT, Bootstrapped likelihood ratio test

**Fig. 1:**
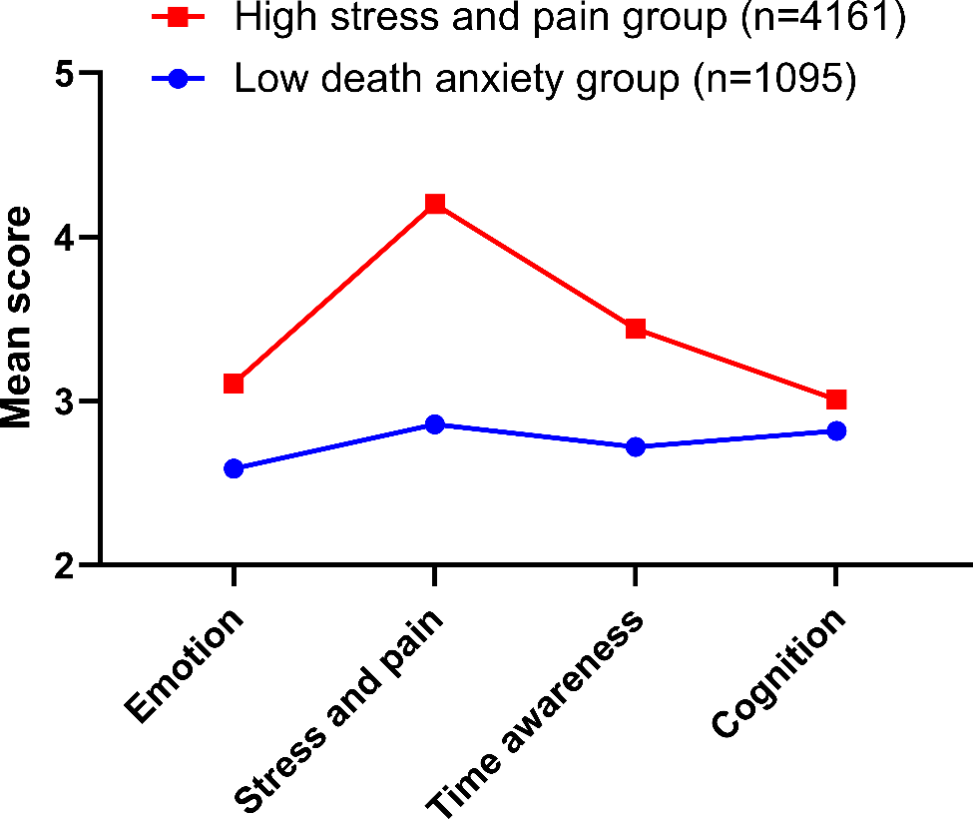
Average score of items in four dimensions of death anxiety of two potential categories

Figure 1 depicted the average scores of items in each dimension of death anxiety classified by the two potential categories. The x-axis represents the four items of CT-DAS, and the y-axis represents the probability of the conditioned reaction. The scores of emotion, stress and pain, time awareness and cognition in Class 2 were significantly higher than those in Class 1 (*p* < 0.05), and the average score of items in the dimensions of stress and pain in Class 2 was more than 4, so Class 1 was named low death anxiety group and Class 2 was named high pressure pain group.

### Differences in the latent classes by characteristics

The statistical description for distributions of each demographic group within latent classes was listed in Table 3. Univariate analysis showed that the prevalence of males in the class 2 group was 63.44%, which was significantly lower than that of females at 79.45% (*p < 0.05*). When considering working years, the incidence of the class 2 was 82.70% among those with ≤ 5 years of experience oncology nurses, significantly higher than the group with 6–10 years of experience and above (*p* < 0.001). Moreover, among national or provincial oncology specialist oncology nurses, the prevalence of the class 2 group was 71.14%, which was significantly lower than that of non-specialist nurses (*p* = 0.014). When examining palliative specialty nurses, it was found that non-national palliative care specialty nurses had a higher proportion in the class 2 group compared to national palliative specialty nurses (69.30% vs. 79.39%, *p* = 0.009). Additionally, in terms of marital status, the incidence of class 2 group was 80.93% among married nurses, significantly higher than single or divorced nurses (*p* < 0.001). Nurse assistant or staff nurse demonstrated a significantly higher percentage within the class 2 group compared to those holding positions as charge nurses or nurse managers.(82.51% vs. 75.57%, *p* < 0.001). It was also observed that oncology nurses who had discussions about death with their patients had a significantly lower proportion of the class 2 groups compared to those who had not (77.70% vs. 81.42%, *p* = 0.001). Similarly, oncology nurses who received training in hospice-related courses had a lower incidence of the class 2 group compared to untrained nurses (75.81% vs. 80.20%, *p* = 0.001).

**Table 3:** The characteristics of potential categories of death anxiety

Characteristic	Low death anxiety group (n = 1095)	High stress and pain group (n = 4161)	χ2	*p*
**Gender**				
Male	34 (36.56)	59 (63.44)	14.196	< 0.001
Female	1061 (20.55)	4102 (79.45)		
**Age**				
<35	793 (21.55)	2886 (78.45)	3.877	0.144
35–50	279 (19.18)	1176 (80.82)		
>50	23 (18.85)	99 (81.15)		
**Working years**				
<5	299 (17.30)	1429 (82.70)	31.105	< 0.001
5–10	418 (20.56)	1615 (79.44)^a^		
>10	378 (25.28)	1117 (74.72)^ab^		
**Oncology nursing specialist**				
Yes	43 (28.86)	106 (71.14)	5.989	0.014
No	1052 (20.60)	4055 (79.40)		
**Palliative care nursing specialist**				
Yes	35 (30.70)	79 (69.30)	6.881	0.009
No	1060 (20.61)	4082 (79.39)		
**Marital status**				
Single or divorced	369 (25.47)	1080 (74.53)	26.03	< 0.001
Married	726 (19.07)	3081 (80.93)		
**Nurse position**				
Nurse assistant or staff nurse	476 (17.49)	2246 (82.51)	38.33	< 0.001
Charge nurse or nurse manager	619 (24.43)	1915 (75.57)		
**Experience of discussing death**				
Yes	711 (22.30)	2478 (77.70)	10.51	0.001
No	384 (18.58)	1683 (81.42)		
**Attended the Palliative training Course**				
Yes	299 (24.19)	937 (75.81)	11.046	0.001
No	796 (19.80)	3224 (80.20)		
**Hospital classification**				
Tertiary hospital	767 (20.70)	2939 (79.30)	0.143	0.705
Secondary and Primary hospital	328 (21.16)	1222 (78.84)		
**Religious beliefs**				
Yes	82 (18.18)	369 (81.82)	2.103	0.147
No	1013 (21.08)	3792 (78.92)		

Note:The Bonferroni method was used to adjust the P value for comparison between two groups. ^a^ was compared with the working years < 5 years, *P* < 0.05; ^b^ was compared with the working years 5–10 years, *P* < 0.05

The total scores of PCQN and FATCOD in Class 1 were higher than those in Class 2 (*p* < 0.05), and the differences in PCQN and FATCOD scores between the two groups were statistically significant, as shown in Table 4.

**Table 4:** The total scores of PCQN and FATCOD-B-C of two latent categories of death anxiety

Characteristic	Low death anxiety group (n = 1095)	High stress and pain group (n = 4161)	t	*p*
PCQN	9.65 ± 3.23	9.22 ± 2.55	4.141	< 0.001
FATCOD	103.05 ± 11.12	101.39 ± 10.7	4.538	< 0.001

The results of multi-classification logistic regression analysis (Table 5) showed that, compared with the low death anxiety group, the influencing factors of high stress pain group were shorter working years, non-national or provincial oncology nursing specialists, non-national palliative care specialists, never discussing the topic of death with patients or family members, no palliative care related training, PCQN and FATCOD scores (*p* < 0.05).

**Table 5:** Multivariate logistic regression analysis of death anxiety in high stress and pain group

Characteristic	β	SE	Wald χ2	P	OR (95%CI)
**Working years**					
<5					1.0 (reference)
5–10	-0.225	0.085	7.059	0.008	0.798 (0.676, 0.943)
>10	-0.490	0.088	30.967	< 0.001	0.613 (0.516, 0.728)
**Oncology nursing specialist**					
Yes	-0.407	0.187	4.740	0.029	0.666 (0.462, 0.960)
No					1.0 (reference)
**Palliative care nursing specialist**					
Yes	-0.483	0.210	5.310	0.021	0.617 (0.409, 0.930)
No					1.0 (reference)
**Level of nursing job**					
Junior level	0.431	0.069	38.897	< 0.001	1.539 (1.344, 1.763)
Medium level and Senior level					1.0 (reference)
**Experience of discussing death**					
Yes	-0.227	0.072	10.051	0.002	0.797 (0.692, 0.917)
No					1.0 (reference)
**Attended Palliative training Courses**					
Yes	-0.260	0.078	11.050	0.001	0.771 (0.661, 0.899)
No					1.0 (reference)
**Total PCQN Score**	-0.049	0.014	12.972	< 0.001	0.952 (0.926, 0.978)
**Total FATCOD Score**	-0.011	0.003	10.541	0.001	0.989 (0.983, 0.996)

## Discussion

In previous studies, research on death anxiety has mainly focused on patients and their families, with little attention paid to oncology nurses’ experiences. In this study, two potential categories of oncology nurses’ death anxiety were identified by LCA, namely, low death anxiety group and high-stress and pain group. The results demonstrate that there are differences in the way oncology nurses’ death anxiety changes throughout the process of caring for dying patients. Among the 5256 oncology nurses surveyed, a small proportion (20.83%) belonged to the low death anxiety group, while the majority (62.90%) belonged to the high-stress and pain group, indicating that most nurses are uncomfortable with death. Frequent exposure to death and dying patients can negatively affect nurses’ emotional well-being and exacerbate their death anxiety [29]. Therefore, oncology nurses need to be adequately prepared to manage their emotions and anxiety when caring for dying patients.

This study examined the relationship between palliative care-related training and death anxiety among oncology nurses. Results showed that oncology nurses who had received such training, including those from palliative care and oncology nursing specialists, had lower rates of death anxiety and a higher likelihood of being classified into the low death anxiety group. These findings are consistent with previous research by Etafa indicating that educational interventions can effectively reduce death anxiety among nurses [30]. In China, however, palliative care is still in its early stages, and healthcare professionals in the field lack training, knowledge, and awareness [31, 32]. Effective communication skills training, which is a crucial component of a palliative care education program, was identified in our previous study as being particularly important in breaking bad news to patients and their families [21]. Nurses working in palliative care often face complex situations that require sensitivity, empathy, and clear communication skills. Without proper training and support, they may struggle to handle these situations appropriately, which can increase their death anxiety. Palliative care-related training can help nurses develop skills such as active listening, empathy, and effective communication strategies, which they can then apply in their practice and reduce their death anxiety.

In a multicultural society, it is necessary to develop a plan that aligns with the cultural background to address cultural norms surrounding death, particularly given that communication skills training in palliative care often has a Western-centric perspective and may not take cultural differences into account [33]. Therefore, it is essential to understand the local culture and improve nurses’ cultural competence to reduce their death anxiety and improve patient care and quality. The knowledge gained from death education provided in palliative care-related training can help oncology nurses understand death scientifically and objectively, reduce their fear or eliminate it, and bring about changes in their behavior, attitude, and empathy, ultimately reducing death anxiety and improving the quality of nursing care [11, 34].

China has a long-standing taboo surrounding death and dying, where discussing it openly is considered impolite or even forbidden. This cultural attitude has had a significant impact on end-of-life care practices. However, there have been efforts to address this taboo, such as the Chinese government implementing policies to promote end-of-life care and education and increased public discussion about death and dying. There are also grassroots organizations working to improve end-of-life care practices and raising awareness about the importance of discussing death and dying openly. Despite these efforts, the taboo surrounding death and dying in China remains a complex cultural issue with significant implications for end-of-life care practices and the experiences of dying patients and their families. More education and awareness-building efforts are necessary to promote open discussions about death and dying and to improve the quality of end-of-life care in China.

The results showed that the scores of nurses’ palliative care attitude in the low death anxiety group was higher than that in the high-pressure and pain group, which was related to the increased stress faced by those nurses caring for dying patients. While nurses and other healthcare professionals may have positive intentions to provide care of the highest quality for patients facing death, they are directly or indirectly affected by the suffering of patients or family members when interacting with dying patients. They may develop fear related to death, which can negatively impact their attitude towards providing care [35]. On the contrary, nurses with a lower fear of death, who have less fear of death and higher acceptance of death, have a more positive attitude towards patients facing sudden death [32]. This suggests that we can help nurses change their attitudes towards palliative care and improve the quality of palliative care by decreasing their death anxiety.

This study also found that oncology nurses with lower scores in palliative care knowledge were more likely to belong to the high death anxiety group. In the face of dying patients, oncology nurses will be caught off guard due to a lack of knowledge in the field of palliative care and a lack of preparation for death and dying, which may lead to anxiety [11]. The knowledge and attitudes of oncology nurses towards palliative care have a significant impact on their level of death anxiety when caring for dying patients. The two variables of palliative care attitude and knowledge are usually regarded as interrelated factors [36, 37]. However, few studies explore the potential relationship among them. The lack of knowledge and preparation may hinder oncology nurses from providing high-quality care and increase stress levels [38]. Nurse managers can provide education and training programs to help oncology nurses develop coping strategies and address any misconceptions or negative attitudes towards death and palliative care. By addressing these issues, oncology nurses can approach the care of dying patients with confidence and provide compassionate, effective care.

In addition, our results showed that the longer the years of working, the lower the level of death anxiety among oncology nurses, which is consistent with the study by foreign scholars [39]. On one hand, senior oncology nurses often participate in the care of critically ill patients. They have a wealth of experience and a better understanding of the suffering endured by patients with serious illness. They usually understand the regrets associated with the deaths of patients who have long-term, life-limiting conditions that cannot be successfully cured. However, those with less experience and who are newly qualified may need more support and mentorship when caring dying patients. With increased contact and care of dying patients, oncology nurses are more likely to let go of feelings of helplessness and burden associated with death. Therefore, they are less easily affected by the negative impact of death.

### Limitations

Several limitations of this study should not be ignored. First, the representativeness and generalizability of the study findings are limited because convenient samples were used, and all participants were from Jiangsu province, which restricts the universality of the study population. Second, our study relied solely on self-reported data, so there may be potential biases due to social expectations. Third, the results of this study indicated that women were more likely to experience death anxiety compared to men, which may be attribute to the significant gender imbalance in this study.

## Conclusions

The death anxiety experienced by oncology nurses can be categorized into two groups: low death anxiety and high-stress and pain. Being Female, having fewer years of work experience, being married, holding only a primary professional title, not being nationally or provincially certified as a oncology nursing specialists, and oncology nurses who have not discussed the topic of death with patients or their family members, and have not received training courses related to palliative care- have are more likely to experience death anxiety.

Based on the conclusions, here are two main clinical implications to consider for nurse managers. First is to provide targeted support and interventions for the high-stress pain group of oncology nurses who exhibit higher levels of death anxiety. This may include stress management programs, counseling services, and support groups tailored to their unique challenges and anxiety. Another implication is to enhance palliative care training programs for oncology nurses, focusing on communication skills, symptom management, and psychosocial support. By implementing these two main clinical implications, healthcare organizations can support oncology nurses in managing their anxiety and enhance their ability to provide optimal care to patients.

## Data Availability

The datasets produced and/or analyzed during this study cannot be made publicly accessible due to ethical restrictions imposed by the Women’s Hospital of Nanjing Medical University. However, interested parties may obtain the data from the corresponding author upon reasonable request.

## List of abbreviations

aBIC: Sample-size adjusted BIC
AIC: Akaike information criterion
BLRT: Bootstrapped likelihood ratio test
CT-DAS: The Chinese version of the Templer Death Anxiety Scale
FATCOD-B-C: Chinese version of the Frommelt Attitude Toward Care of the Dying scale
LCA: Latent Class Analysis
LMR: Lo-Mendell-Rubin
PCQN-C: Chinese version of The Palliative Care Quiz for Nursing
PCQN: The Palliative Care Quiz for Nursing
